# The Role of Elevated Midlife Blood Pressure on Cognition and White Matter Hyperintensities

**DOI:** 10.1002/alz70860_107355

**Published:** 2025-12-23

**Authors:** Anisa Dhana, Charles DeCarli, Klodian Dhana, Neelum T. Aggarwal, Kyle Dennis, Denis A Evans, Kumar B Rajan

**Affiliations:** ^1^ Rush Institute for Healthy Aging, Chicago, IL, USA; ^2^ Rush University Medical Center, Chicago, IL, USA; ^3^ University of California, Davis School of Medicine, Sacramento, CA, USA; ^4^ Alzheimer's Disease Research Center, University of California Davis, Sacramento, CA, USA; ^5^ Rush Alzheimer's Disease Center, Chicago, IL, USA; ^6^ Rush University Medical Center, hicago, IL, USA; ^7^ University of California Davis, Davis, CA, USA

## Abstract

**Background:**

Elevated blood pressure has been associated with low cognitive scores and increased white matter hyperintensity volume in older adults. However, it remains unclear whether these associations begin in midlife.

**Methods:**

This study included 439 individuals aged 40 to 64 from the Parent Offspring Resilience and Cognitive Health Study (PORCH), a longitudinal study in Chicago since 2019. Trained research assistants measured blood pressure using a standardized sphygmomanometer. Cognition was assessed by validated and established cognitive tests, including delayed and immediate logical memory tests, category fluency for animals, fruits, and vegetables, and digit forward and backward tests. White matter hyperintensities were evaluated through a combination of fluid‐attenuated inversion recovery and 3‐dimensional T3 magnetic resonance imaging scans utilizing a modified Bayesian probability structure rooted in a previously published histogram fitting method. Linear regression analysis assessed the relationship between systolic and diastolic blood pressure, global cognition, and white matter hyperintensity volumes in models adjusted for age, sex, race, and education.

**Results:**

Of the 439 individuals included in this analysis, 57% (*n* = 249) were females, and 32% (*n* = 139) were Black participants. The mean (SD) age was 56.8 (5.0) years, with an average systolic blood pressure of 125 mmHg and diastolic blood pressure of 80 mmHg. Elevated systolic blood pressure was associated with increased white matter hyperintensity volume but not with cognitive scores at the baseline. For a 10 mmHg increase in SBP, white matter hyperintensities volumes were 1.19 (*p*‐value=0,0267) units higher. Diastolic blood pressure showed no significant association with white matter hyperintensity volumes.

**Conclusion:**

Elevated systolic blood pressure in midlife was associated with higher white matter hyperintensity volumes, suggesting that the adverse effects of blood pressure on brain structure may start in midlife.

